# Aggregated Clumps of Lithistid Sponges: A Singular, Reef-Like Bathyal Habitat with Relevant Paleontological Connections

**DOI:** 10.1371/journal.pone.0125378

**Published:** 2015-05-27

**Authors:** Manuel Maldonado, Ricardo Aguilar, Jorge Blanco, Silvia García, Alberto Serrano, Antonio Punzón

**Affiliations:** 1 Centro de Estudios Avanzados de Blanes (CEAB-CSIC), Blanes, Girona, Spain; 2 Oceana, Madrid, Spain; 3 Instituto Español de Oceanografía, Centro Oceanográfico Santander, Santander, Spain; ufrj, BRAZIL

## Abstract

The advent of deep-sea exploration using video cameras has uncovered extensive sponge aggregations in virtually all oceans. Yet, a distinct type is herein reported from the Mediterranean: a monospecific reef-like formation built by the lithistid demosponge *Leiodermatium pfeifferae*. Erect, plate-like individuals (up to 80 cm) form bulky clumps, making up to 1.8 m high mounds (1.14 m on average) on the bottom, at a 760 m-deep seamount named SSS. The siliceous skeletal frameworks of the lithistids persist after sponge death, serving as a complex 3D substratum where new lithistids recruit, along with a varied fauna of other sessile and vagile organisms. The intricate aggregation of lithistid mounds functions as a “reef” formation, architecturally different from the archetypal "demosponge gardens" with disaggregating siliceous skeletons. *Leiodermatium pfeifferae* also occurred at two additional, close seamounts (EBJ and EBS), but, unlike at SSS, the isolated individuals never formed accretive clumps. The general oceanographic variables (temperature, salinity, dissolved nutrients, chlorophyll, and oxygen) revealed only minimal between-seamount differences, which cannot explain why sponge abundance at SSS is about two orders of magnitude higher than at EBJ or EBS. Large areas of the dense SSS aggregation were damaged, with detached and broken sponges and a few tangled fishing lines. Satellite vessel monitoring revealed low fishing activity around these seamounts. In contrast, international plans for gas and oil extraction at those locations raise serious concerns over the need for protecting urgently this unique, vulnerable habitat to avoid further alteration. Modern lithistids are a relict fauna from Jurassic and Cretaceous reefs and the roots of the very genus *Leiodermatium* can be traced back to those fossil formations. Therefore, understanding the causes behind the discovered lithistid aggregation is critical not only to its preservation, but also to elucidate how the extraordinary Mesozoic lithistid formations developed and functioned.

## Introduction

Sponges are long recognized as important members of many marine communities. Under circumstances that remain poorly understood, they are able to aggregate in unusually high abundances, often becoming major habitat builders. Aggregated sponges offer substrate for attachment, shelter, and feeding of other organisms, also modify benthic boundary layers, and impact significantly local and regional fluxes of organic carbon and dissolved inorganic nutrients [1–8].

During the past two decades the advent of deep-sea exploration using Remote Operated Vehicles and submersibles has uncovered extensive aggregations at deep-shelf, bathyal, and abyssal bottoms, often being referred to as "sponge gardens" or "sponge grounds" [4–5,9–15]. Among the several types of deep-water discoveries, the most shocking one were accumulative aggregations of hexactinellid sponges at the shelf-slope transition (150 to 260 m deep) off the coast of British Columbia, Canada [16]. The aggregations consist of 3 species of hexactinellids characterized by a massive skeleton of fused silica pieces (informally referred to as "dictyonine skeletons"), which dissolves very slowly [17] and provides a substratum on which new sponge individuals settle. This accretive process results in up to 20 m high reef formations. These sponge reefs are thought to constitute a living formation analogous to extinct Mesozoic sponge reefs [16,18–19]. Interestingly, in Paleozoic (542–252 mya) and Mesozoic (252–65 mya) oceans, two phylogenetically unrelated types of siliceous sponges ("dictyonine" hexactinellids and "lithistids") were major builders of reefs and sponge aggregations (i.e. sponge facies) in reef environments. These two ancient sponge archetypes have survived to Recent time, being the "dictyonines" classified into the modern subclass Hexasterophora of the class Hexactinellida and the "lithistids" as part of the class Demospongiae. Both sponge types share a skeletal feature: a massive siliceous skeleton in which the original, discrete pieces (spicules) become fused or articulated into a rigid silica framework. This skeletal hypersilicification has no phylogenetic value, being well established that rigid silica skeletons arose independently both in several orders within hexasterophorid hexactinellids and in different demosponge orders. Therefore, the terms "dyctionine" and "lithistid", rather than respectively reflecting a particular phylogenetic assignation within Hexactinellida and Demospongiae, informally allude to a skeletal condition (i.e., fused or articulated, rigid skeletons) of some hexactinellids and demosponges.

Hexactinellids and lithistids commonly co-occurred as reef builders on Late Jurassic (163–145 mya) and Early Cretaceous (145–100 mya) shelves, particularly at the "European" coasts of the Tethys Sea [20–21]. Those Mesozoic sponge reefs became extinct. However, from the joint analysis of the fossil record in the Paleogene (65 to 23 mya) and present-day distributions, it has been concluded that recent dictyonine hexactinellids and lithistids are relict faunas from the Cretaceous Tethyan assemblages [21–24]. These survivors, now inhabiting mostly bathyal bottoms and/or caves, were thought not to retain their ability to produce silica reef constructions any longer, until, to everyone's surprise, the living equivalent to the extinct Mesozoic hexactinellid reefs was discovered at British Columbia. It has taken over a decade to elaborate a comprehensive physical description of that unique silica reef habitat and its oceanographic setting [18–19,25–27], and almost two decades to realize about its role as an essential habitat in need of protection [28]. Only very recently have investigations focused on the functional implications that the phenomenal nutrient fluxes across the hexactinellid reef may have in the benthic pelagic-coupling at the regional scale [17,29].

Here we are reporting on the discovery of another extant reef-like aggregation with relevant paleontological connections. It is not built by hexactinellids, but by lithistid demosponges, the one other existing type of relict sponges having a massive skeleton made by a rigid silica framework. The biology and ecology of present-day lithistid fauna remain poorly known. It can be summarized: 1) that these sponges are virtually absent from high latitudes (only 1 species known); 2) that a few species grow in shallow caves at tropical and temperate latitudes; and 3) that most species occur at bathyal depths, typically on rocky slopes and seamounts, and rarely deeper than 1700 m, although some abyssal species are known [22,30–32]. Most of the scarce biological data come from shallow caves [33–34], with information on bathyal lithistid assemblages being poorer and more fragmentary. The richest bathyal lithistid fauna known in modern oceans is probably that described from a South-Pacific chain of seamounts (200 to 700 m deep) at the Norfolk Ridge in New Caledonia [22,24,31,35–37]. That lithistid fauna has been interpreted systematically as relict from the Cretaceous, with elevated species diversity and abundance values comparable only to those in fossil assemblages from the Mesozoic.

The newly discovered lithistid aggregation, by its monospecific condition and its architectural organization, has no equivalent among the present-day lithisthid assemblages from New Caledonia or elsewhere. We are aware that the current report, because of logistic and gear limitations, does not provide a full-detail habitat characterization and that much multidisciplinary work is still required for a comprehensive understanding of the extension, oceanographic setting, and functioning of this newly discovered type of sponge formation. Nevertheless, given the offshore location and depth of the discovered habitat, it is likely that further investigations will not be conducted before new research expeditions are arranged, in years or decades. Therefore, the present study has to be understood as an initial communication aiming to inform about the existence and location of this singular, vulnerable habitat in order to attract scientific attention and, more importantly, awareness for its immediate preservation.

## Materials and Methods

### Study site and sampling

**Fig 1:**
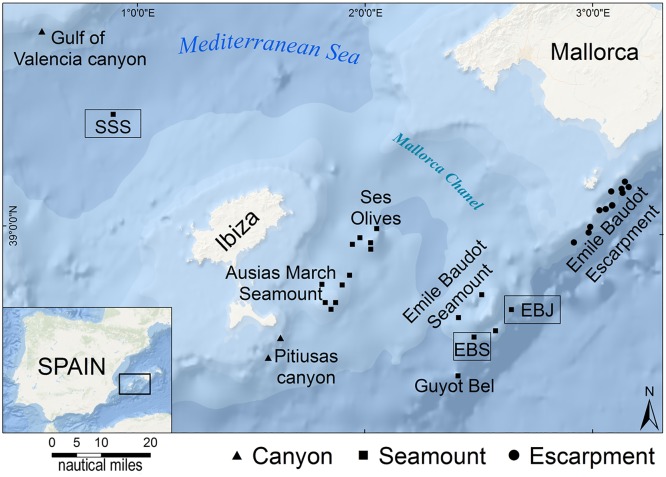
Study site location. The inset shows the location of the Western Mediterranean surveyed area. The large map indicates the location of the 32 ROV transects. The framed squares (Stone Sponge Seamount = SSS; Emile Baudot Jr. Seamount = EBJ; Emile Baudot South Seamount = EBS) indicate the 3 seamounts reported in this study where the lithistid sponges grew abundantly.

During 2013 and 2014, two research expeditions were carried out to investigate the fauna associated with deep bottoms around the Balearic Islands (Spain, Western Mediterranean; Fig 1). The study was conducted using the "Oceana Ranger" catamaran, a 71-feet sailing boat re-adapted to use a Remotely Operated Vehicle (ROV), but unfortunately lacking laboratories and any type of geological and oceanographic equipment. Bottom inspection was carried out using a Saab Seaeye Falcon DR ROV equipped with a HDV camera of 480 TVL with Minimum Scene Illumination 2.0 LUX (F1.4), Pick Up Device ½” CCD, Image Sensor, and spherical ½ of 3.8 mm and wide angle lens. A total of 32 transects were run at depths ranging from 100 to 1,000 meters, particularly focusing on escarpments, canyons, and seamounts. In this study, we are reporting on particular findings at 3 seamounts around the Balearic Islands, one located in the Gulf of Valencia-Ibiza Channel and two others south of the Mallorca Channel (Fig 1). The former seamount, herein baptized as "Stone Sponge" (SSS; 39°21.345’N, 000°51.417’E), rises from a 1,300 m-deep surrounding bottom to a minimum depth of 730 m below ocean surface. The two others are small pinnacles relatively close to each other on the east and south sides, respectively, of the Emile Baudot seamount (Fig 1), being herein named as Emile Baudot Junior (EBJ; 38°42.879’N, 002°36.254’E; top located at a depth of 495 m) and Emile Baudot South (EBS; 38°37,3580’N—002°26,2047’E; top located at a depth of 600 m). The surveyed bottom depths at SSS ranged from 730 to 890 m, at EBJ from 495 to 735 m, and at EBS from 615 to 665 m.

Except for limited ROV collections, most faunal data derived from video transects and photography. All digital video and still images were geo-referenced using the USBL data from the ROV and analyzed subsequently in the laboratory. The size of the collected organisms, sponge portions included, was also used as additional scaling reference to estimate dimensions of individuals and small-scale distances of interest.

### Faunal identification

Whenever possible, representatives of the dominant benthic fauna were collected using the ROV’s arm to complement the visual information acquired by the ROV surveys. The sampling area—located in Mediterranean’s high seas, 12 nautical miles outside of territorial waters—is a non-protected zone where specific collection permits are not required under either international or national legislation. Likewise, the conducted research did not involve impact upon protected species listed under any international or national law.

Samples of the lithistid sponge were preserved in 70% ethanol upon collection. Photographs of body parts of fixed individuals were taken through an Olympus SZX2 dissecting microscope connected to a ProgRes C7 digital camera.

For the skeletal description of the dominant sponge species, tissue was cleaned in boiling nitric acid, rinsed in distilled water, dehydrated in 70% and 100% ethanol for 15 minutes at each step, and dried at 50°C. For the light microscopy study, dried skeleton samples were mounted on glass slides and examined under an Olympus IX51 inverted microscope connected to a digital camera. Additionally, uncoated, acid-cleaned samples were mounted on aluminum stubs and studied through a HITACHI TM3000 Scanning Electron Microscope (SEM).

Vouchers of the collected lithistid material have been stored at the National Museum of Natural History of Madrid (NMCN—CSIC).

### General oceanographic setting

The general oceanographic conditions around the sites of interests were characterized using data available from the Mediterranean Hydrographic and Biological Data Archiving Project (http://modb.oce.ulg.ac.be/backup/medar), exploring particularly the -800 m isobath of the Balear Sea over a year cycle. The magnitudes of between-site differences in concentration of dissolved nutrients (silicate, ammonium, nitrate, nitritie, phosphate), chlorophyll a, dissolved oxygen, temperature, and salinity have been examined in order to assess their potential relevance in the development and/or maintenance of the sponge aggregations.

Because of the lack of adequate geological and coring gear during the cruises, the geomorphological setting at and around the sponge aggregations could not be herein characterized.

### Vulnerability to deep-sea human activities

To assess the risk posed by the fishing fleet on the sites of interest, we examined the spatial distribution of demersal fishing effort by the Spanish fishing fleet, which is the only one operating in the area. Satellite information on vessel monitoring system (VMS) was made available to us by the Spanish Ministry of Agriculture, Food, and Environment (MAGRAMA) for the period 2007 to 2010. The VMS database provides information on the position (at 2h intervals), speed, and course of each vessel larger than 15 m in length. To focus on relevant fishing activity exclusively, we filtered out from the global VMS database irrelevant information relative to navigation periods, time in harbor, etc. For this data filtering [38–39], 1) the time interval and the Euclidean distance between successive signals was used; 2) the average vessel speed was calculated from the interval between successive signals; 3) vessels with less than ten signals in a year were removed; and 4) signals recorded within a distance of three miles or less from the closest fishing harbor were eliminated. The type of fishing gear used by the monitored Spanish vessels was found out by linking the VMS data to national logbook data using the vessel identifier, date, and landing species. Based on the frequency distribution of average speeds, a working range for each fishing gear was defined, and all signals out of the working range were eliminated [39]. Finally, the fishing activity of 1,045 vessels was considered in the analysis, generating estimates of fishing effort (fishing hours) with a 5' (i.e., 5 nautical mile) grid resolution. Because this approach does not include every demersal fishing activity but only those by vessels emitting VMS data, we are expressing fishing activity (pooling together otter trawls, set gillnets, set longlines, and traps) as relative fishing effort (%), calculated on the maximum effort obtained in a spatial cell.

To further identify potential threats and damaging human activities, we examined the public records available at the Spanish Ministry of Industry, Energy and Tourism for information on enterprises developing deep-sea activities at the Balearic Sea or having recently requested permissions to do so. Using that information, which is published in the Boletin Oficial del Estado (BOE) and at the Ministry the web page (http://www6.mityc.es/aplicaciones/energia/hidrocarburos/petroleo/exploracion2014/mapas/inicio.html), we have elaborated maps summarizing the threat posed by planned seismic surveys and hydrocarbon extraction on the 3 studied seamounts.

## Results

### Description of the lithistid aggregation

**Fig 2:**
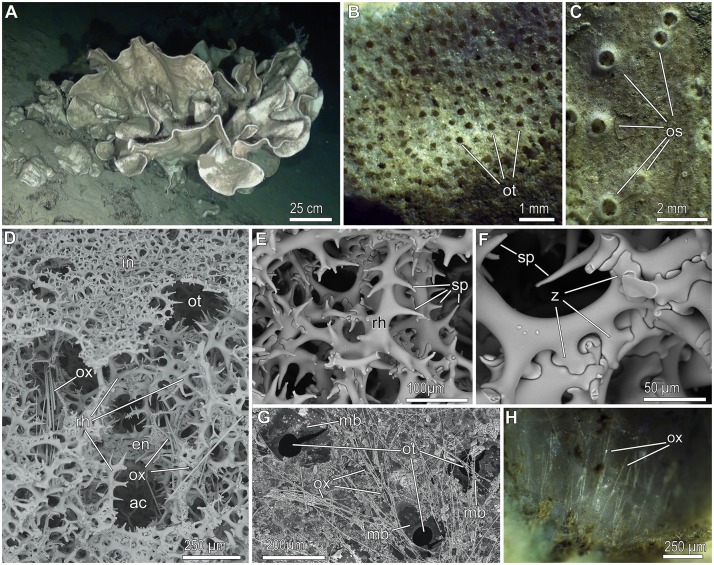
Features of *Leiodermatium pfeifferae*. (**A**) *In situ* view of an isolated, small group of individuals. (**B**) Detail of the convex, inhalant side of a dried individual, showing punctiform ostioles (ot). (**C**) Detail of the concave, exhalant side, showing small elevated oscules (os). (**D**) General view of the framework of spiny rhizoclone desmas. At the internal (en = endosome) region of the body plate, packs of oxeas (ox) and the aquiferous canals (ac) run from side to side of the sponge, passing through the network of desmas (rh). Note that at the inhalant side (in), desmas are thinner and smaller, making a more denser network that functions as a dermal skeleton among the ostia (ot). (**E**) Detail of rhizoclones (rh) around an aquiferous canal, showing large spines (sp). (**F**) Detail of contacts (z = zygoses) between adjacent desmas. (**G**) SEM view of the inhalant side of a dried specimen, showing abundant hispidating oxeas (ox) and ostia (ot) provided with a poral membrane (mb) that allows these pores to be closed if required. (**H**) Detail of hispidating oxeas forming a subterminal fringe at the free edge of the plates.

In 3 out of the 32 ROV transects, a high abundance of the lithistid demosponge *Leiodermatium pfeifferae* (Carter, 1873) was found. This is a species never recorded before in the Mediterranean (Fig 2A; see section on "Taxonomic Remarks"). In all 3 cases, the sponge populations were located around the top of seamounts (namely, SSS, EBJ, and EBS; Fig 1). The sponge individuals grew with a foliose body shape, forming erect, contorted plates that were 0.3 to 0.9 cm thick, up to about 80 cm in height, and up to 100 cm across (Fig 2A). On the convex side, 0.1–0.2 mm wide, in-going orifices (ostia) were scattered at high density (Fig 2B), forming a feeding (inhalant) surface through which the water enters the sponge carrying food (i.e., picoplankton) and oxygen. Scattered on the concave side of the plates, there were slightly elevated, 0.5–0.6 mm wide, out-going orifices (oscula; Fig 2C), through which seawater is expelled from the sponge body after being depleted from picoplankton and oxygen, also carrying the metabolic wastes away.

**Fig 3:**
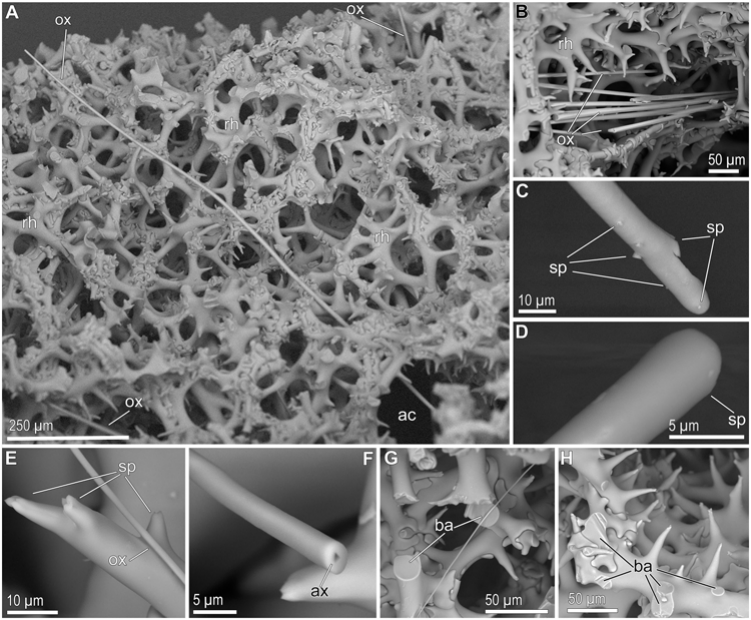
Skeletal details of *Leiodermatium pfeifferae*. (A) General view of the desma (rh) network, with oxeas (ox) and aquiferous canals (ac) passing through. (B) Pack of oxeas (ox) running through the desma (rh) network. (C-D) Detail of the round, spiny (sp) ends of the thickest strongyloxeas occurring in the marginal fringe. (E) Detail of a very thin oxea (ox) on the typical multifurcated spine (sp) of a rhizoclon desma. (F) View of the triangular axial canal (ax) at the core of a broken oxea. (G-H) Broken arms (ba) of desma showing no internal axial canal.

The sponges were hard as stones, because their body contains a massive skeletal framework made of articulated/fused siliceous spicules, the rhizoclone desmas (Figs 2D–2F and 3A). The rigid skeleton of these spiny and irregularly-shaped desmas is complemented with long (up to 1.9 mm), very thin (1 to 8 μm), flexuous, needle-like spicules, called oxeas (Figs 2D, 2G, 2H, and 3A). Oxeas run from one side to another of the sponge body, passing through the network of desmas (Figs 2D, 3A, 3B, and 3G), and piercing out the body surface at the inhalant side (Fig 2G) and also at the margin of the plate, where they form an hispidating fringe (Fig 2H). The mass of the siliceous skeleton is so important in these sponges that it represents about 95± 2% of the body dry weight. As in the case of the Canadian hexactinellid reefs [17], the massive lithistid skeletons do not disaggregate and appear to persist in place for long periods after sponge death.

**Fig 4:**
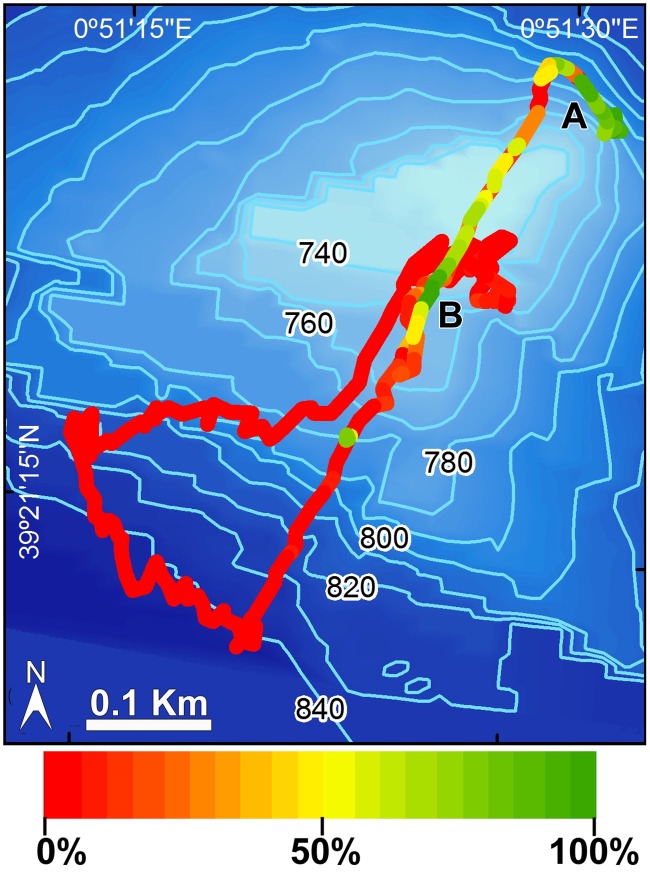
ROV track at the Stone Sponge Seamount (SSS). Substrate coverage by lithistid sponges along the track is indicated by the color scale. A and B sites refer to the two areas where the lithistid reef was intersected by the track.

The densest and largest aggregations of *L*. *pfeifferae* occurred at SSS (S1–S3 Videos) and were discovered when a 2,260 m long and 1.5 wide ROV transect intersected the sponge aggregation at 2 different sites (sites A and B in Fig 4). We do not know the exact extension of the sponge field beyond the narrow ROV pathway. Preliminary explorations at site B by running random trajectories perpendicular to the main ROV pathway revealed no additional extensive sponge aggregation (Fig 4). Nevertheless, a more detailed, systematic bottom exploration at this seamount is required to establish the real extension of the sponge aggregation, particularly at the isobath of site A, where the lithistid formation is suspected to extend as a subterminal crown around the seamount.

**Fig 5:**
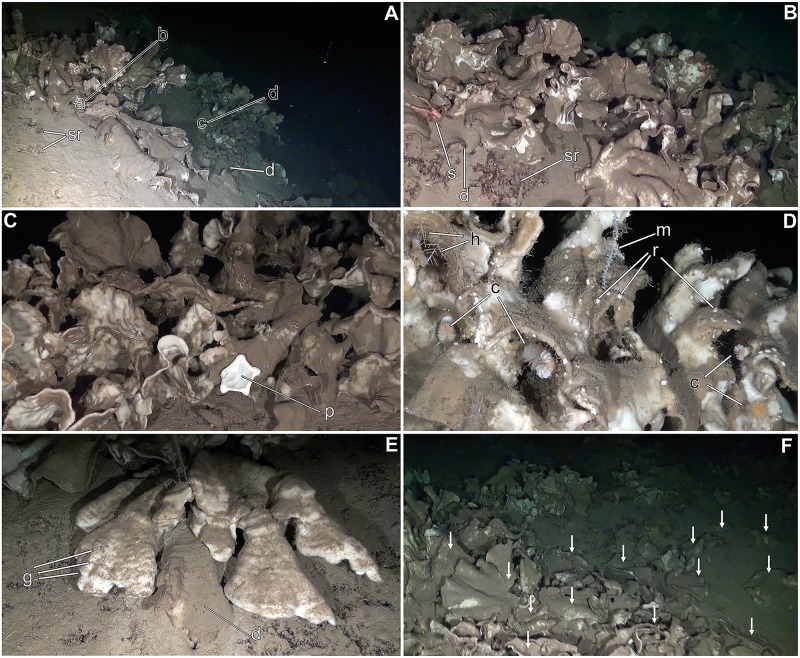
Views of lithistid reef at Stone Sponge Seamount (SSS). (**A-B**) General view of the lithistid mounds, showing intertwined growth and a complex 3D structure. Translucent "a-b" and "c-d" lines indicate sponge clumps measuring respectively 118 cm and 94 cm, relative to sediment bottom. Note the occurrence of dead sponges (d) and bare skeletal remains (sr) buried in the sediment. A squat lobster (s) is also shown. (**C**) View of sponge plates, showing how the sediment rain accumulates on the convex (in = inhalant) side, while the concave (ex = exhalant) side has no silt. The abundant hispidating spicules on the inhalant side appear to facilitate the accumulation of sediment on the sponges. Note the presence of the starfish *Peltaster placenta*, thought to be also a suspension feeder. (**D**) Common invertebrates growing on the lithistid are hydroids (h), the alcyonacean octocoral *Muriceides lepida* (m), and the scleractinian coral *Desmophyllum dianthus* (c) with abundance of newly settled recruits (r). (**E**) Group of lithistid individuals laying on the sediment. Note the superimposing structure of the clump. One of the individuals is already dead (d), buried in sediment. On the other sponges, growth marks (g) are seen on the bodies, probably reflecting periodical pulses of food and silicate in the sponge habitat. (**F**) Aggregation area seriously damaged, with large sponges broken and laying on the side (arrows) while being buried under the sediment rain.

The exact number of superimposed sponge layers below the sediment remain undetermined from the current, non-coring approach, but two or more sponge layers semi-buried by sediment were often evident (Fig 4A and 4B; S1–S8 Videos). Many individuals grew as large plates, being usually 30 to 50 cm across, with the largest individuals reaching about 1 m across and up to 80 cm in height. These sizes are striking, given that the individuals of this species (and even those of most other species in the genus) have never been described growing larger than 35 cm across and 30 in height. Accretive, clumped growths produce sponge mounds on the bottom (Fig 5A), conservatively estimated to reach a maximum height of 180 cm, being on average 114 ± 35 cm (n = 12). Because at SSS the individuals grew partially superimposed to each other and intertwined (Fig 5), it was difficult to quantify density with accuracy. Tentative counts at the top layer of the sponge formation indicated that densities may range from a single large individual per m^2^ in some areas to about 15–16 in others, with 5 individuals of diverse size per m^2^ being the modal value.

Substrate occupancy at SSS was monitored in more detail for about 330 m^2^ of the sponge bed, revealing that sponges covered from 5% to virtually 100% of bottom within the ROV trail (Fig 4), averaging about 41.6±29.5%. At EBJ and EBS, the lithistid *L*. *pfeifferae* was a common species, but never forming the dense, accretive aggregations found at SSS. For comparative purposes, a 300 m^2^ transect was run at EBS between the sites 38°37.3580’N—002°26,2047’E at 615 m and 38°37.4820’N—002°26.0757’E at 665 m. It resulted in a total count of 50 scattered individuals, averaging a density of 0.1 ± 0.3 ind m^-2^, that is, between one and two orders of magnitude lower than at SSS.

At those bottom areas where substrate coverage by the sponges was not continuous, small patches of blackish rock were occasionally seen, being likely volcanic basalt, which is the main constituent of the Emile Baudot Seamount System, created by Pleistocenic vulcanism [40]. More often, the substrate was exposed to a continuous sediment rain, under which rocks, living sponges and skeletal remains of dead individuals were slowly buried (Fig 5A and 5B). Probably because of the shortage of hard bottom, the body and the skeletal remains of the lithistids were often chosen as settlement substratum by many sessile organisms (Fig 5D; S4 Video), typically hydroids, alcyonid and scleractinian corals, bryozoans, and, more rarely, other sponges. The scleractinian coral *Muriceides lepida* was particularly common at the SSS aggregation, growing on both sponges (Fig 5D) and rock. More importantly, the aggregation of *L*. *pfeifferae* individuals at SSS formed a complex 3D "reef-like" habitat that attracted a diverse vagile fauna dominated by fish, crustaceans and echinoderms, with common occurrence of conger eels, shrimps, squat lobsters, crabs, starfish, and sea urchins (Figs 5B, 5C, and 5D, Table 1; S5 and S6 Videos). The EBJ and EBS bottoms were slightly shallower and less silted than the SSS site, therefore offering a larger diversity of substrata and benthic environments, forming a faceted landscape of sediment-clean and silted rocks, crevices, coral frameworks, cobble beds, tanatocenose of the gryphaeid mollusc *Neopycnodonte zibrowii*, fine-sand bottoms, etc. At these two sites, the gorgonians *Bebryce mollis* and *Nicella granifera* formed gardens, dominating the landscape. The lithistid *L*. *pfeifferae* typically occurred interspersed among the other fauna, but preferentially occupying heavily silted bottom zones that, in turn, appeared to be avoided by the dominant gorgonians. At EBJ and EBS sites, the accompanying sponge community appeared to have a somewhat higher species richness than at SSS, where *L*. *pfeifferae* largely dominated the available hard substratum for sponge settlement. Common accompanying sponge species were *Stylocordila pellita*, *Pachastrella monilifera*, *Thenea muricata*, and *Tetrodictyum tubulosum* (Table 1).

**Table 1:** Accompanying fauna.

TAXA	SSS	EBJ	EBS	TAXA	SSS	EBJ	EBS
PORIFERA				BRYOZOA			
*Cladocroce fibrosa*		X	X	Bryozoa sp.1			X
Demospongiae sp.1		X	X	*Kinetoskias* cf. *smithii*			X
Demospongiae sp.2		X	X	ANNELIDA			
Demospongiae sp.3	X	X		Polychaeta sp.1	X	X	X
Demospongiae sp.4		X		Polychaeta sp.2		X	
Demospongiae sp.5		X		Polychaeta sp.3			X
Demospongiae sp.6		X		MOLLUSCA			
Demospongiae sp.7		X		*Bathypolypus* sp.1	X		
Demospongiae sp.8		X		Solenogastres sp.1	X		
Demospongiae sp.9	X		X	*Spondylus gussonii*		X	X
Demospongiae sp.10			X	*Todarodes sagittatus*	X		
Demospongiae sp.11			X	CRUSTACEA			
Demospongiae sp.12			X	*Ampelisca* sp.1			X
*Farrea* sp.1		X		*Aristeus antennatus*	X	X	
Lithistida sp. 1		X		*Bathynectes maravigna*	X		
*Leiodermatium pfeifferae*	X	X	X	*Geryon trispinosus*	X	X	
*Poecillastra amygdaloides*		X		*Meganycthiphanes norvegica*	X	X	
*Poecillastra compressa*		X		*Munida* spp.	X	X	X
*Pachastrella monilifera*		X	X	Mysidacea spp.		X	X
*Rhizaxinella pyrifera*		X		Pandalidae spp.	X	X	
*Stylocordyla pellita*	X	X	X	*Pagurus* sp.1			X
*Sympagella* sp.1		X	X	*Paromola cuvieri*	X	X	X
*Tretodyctium tubulosum*		X	X	*Plesionika gigliolii*		X	X
*Thenea muricata*			X	*Plesionika martia*	X	X	X
CNIDARIA				ECHINODERMATA			
*Bebryce mollis*		X		*Cidaris cidaris*	X		
*Callogorgia verticillata*			X	Crinoidea spp.		X	
*Caryophyllia calveri*		X		*Leptometra phalangium*		X	X
*Caryophyllia* sp.1	X	X	X	*Peltaster placenta*	X		
*Desmophyllum dianthus*	X			PISCES			
*Hormathia alba*			X	*Chaulodius sloani*	X		
Hydrozoa sp.1		X		*Coelorhynchus caelorhynchus*		X	
Hydrozoa sp.2	X	X	X	*Conger conger*	X		
Hydrozoa sp.3	X			*Epigonus constanciae*		X	
Hydrozoa sp.4	X			*Galeus melastomus*			X
Hydrozoa sp.5			X	*Helicolenus dactylopterus*	X	X	X
*Muriceides lepida*	X			*Hoplostethus mediterraneus*			X
*Nicella granifera*		X	X	*Hymenocephalus italicus*	X		
*Savalia savaglia*		X		*Lampanyctus crocodilus*			X
*Scleranthellia* sp.1		X		*Lepidion eques*	X		
Scyphozoa sp.1	X			*Nezumia aequalis*	X		X
*Solmissus albescens*		X	X	*Phycis blennoides*	X	X	X
*Epizoanthus* sp.1			X	*Polyacanthonotus rissoanus*	X		
Zoanthidae sp.1	X			*Polyprion americanus*		X	

List (presence/absence) of the most common animals identified in association with the lithistid populations during ROV surveys at SSS, EBJ, and EBS.

### General oceanographic setting

**Fig 6:**
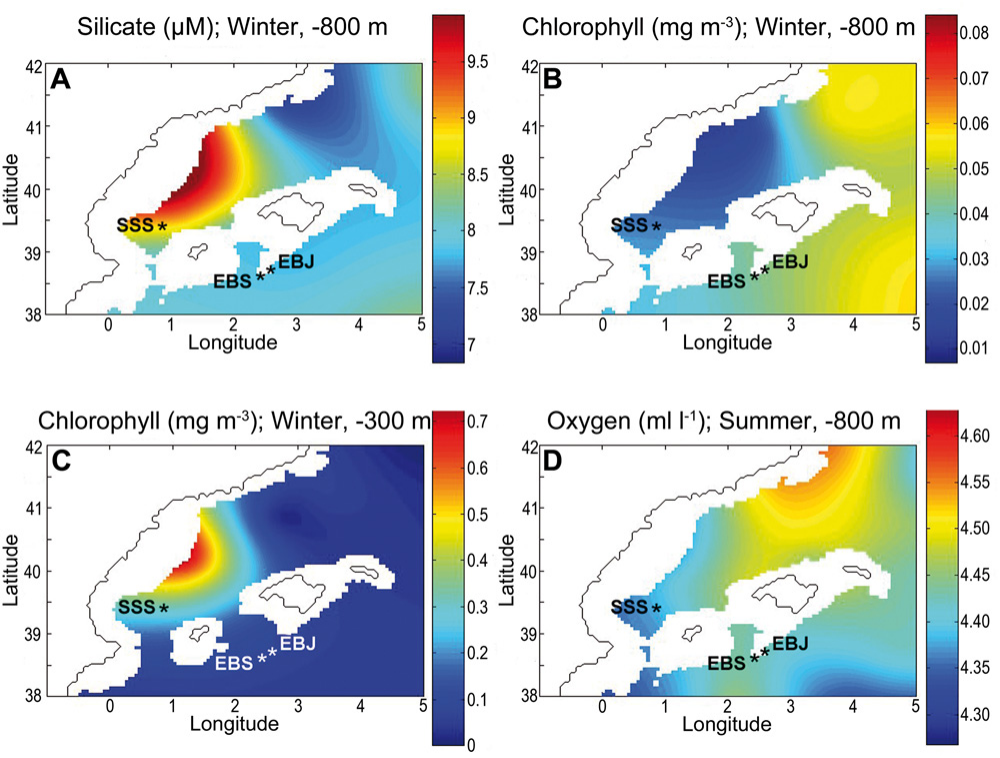
General oceanographic features. (**A**) Silicate concentration (μM) at the -800 m isobath of the Balearic Sea. (**B-C**) Chlorophyll concentration (mg m^-3^) at the -800 m and -300 m isobaths; the latter one indicates the coastal origin of the sinking phytoplankton. (**D**) Oxygen concentration (ml l^-1^) at the -800 m isobath. To illustrate between-mountain comparisons in the various variables of interest, we are depicting only the spatial distribution during the season in which differences between the SSS area and the EBJ-EBS area were the greatest for each variable. In general, between seamount differences were very small over the year cycle, with the winter season causing the larger differences, except for oxygen concentration, which took place in summer.

The mean concentration of silicate, a crucial nutrient used by sponges to build their silica skeleton, ranges from 7.50 to 9.50 ± 0.60 μM over the year at the -800 m isobath of the SSS area, being only 1 to 1.5 μM higher on average than values around the two others seamounts where the sponge occurs in far lower abundance (Fig 6A). The slightly higher values around SSS, which is the seamount closest to mainland, probably reflect the coastal origin of silicate inputs, as suggested by the silicate concentration at the -50 m and -300 m isobaths (not shown). The other dissolved inorganic nutrients (ammonium, nitrate, nitrite, and phosphate) are less significant to the sponge biology, but, likewise, no marked between-seamount differences in their concentration were detected. Nitrate concentration at the -800m isobath remains almost invariable over the year, with similarly low concentrations at both areas (SSS: 7.60 ± 0.25 μM; EBJ-EBS: 7.72 ± 0.25 μM). A similar pattern, but with much lower concentrations, was detected for ammonium (SSS: 0.36 ± 0.27 μM; EBJ-EBS: 0.1 ± 0.27 μM), nitrite (SSS: 0.07 ± 0.03 μM; EBJ-EBS: 0.05 ± 0.03 μM), and phosphate (SSS: 0.43 ± 0.03 μM; EBJ-EBS: 0.42 ± 0.05 μM).

Temperature and salinity values at the -800 m isobath keep quite stable, with negligible between-seamounts differences over the year. The average concentration of Chl *a* at the -800 isobath, which could inform about inputs of organic matter from the euphotic zone, ranges from 0.025 to 0.33 ± 0.01 mg m^-3^ around SSS over the year, being systematically these figures about 0.01 mg m^-3^ lower than the average concentrations at EBJ and EBS sites (Fig 6B). These values are one order of magnitude lower than the chlorophyll concentration measured at the -300 m isobath (Fig 6C) and about 2 orders of magnitude lower than those in the chlorophyll maximum located in the euphotic layer at a depth of 50 m (not shown). Chlorophyll distribution at -300 m illustrates the sinking of coastal phytoplankton bloom, which should deliver about 2 or 3 times more organic matter to SSS than to EBS-EBJ (Fig 6C). Nevertheless, the complex hydrodynamic taking places around and between these deep seamount chains (see Discussion) causes the SSS site to finally receive even lower Chlorophyll inputs than EBS and EBJ seamounts (Fig 6B). Oxygen concentrations, a potential indicator of decomposition of sunk organic matter through bacterial proliferation, confirmed the chlorophyll patterns at -800 m. Oxygen varied minimally around the 3 seamounts, ranging from 4.36 to 4.48 ml l^-1^ over the year cycle, and with between-seamount differences being always smaller than 0.09 ml l^-1^ (Fig 6C).

### Habitat damage and vulnerability

Our ROV survey at SSS documented areas where the lithistid aggregation was seriously damaged, with many detached and broken sponges laying in unnatural position on the bottom while being slowly buried under a lethal sediment rain. From S7 and S8 Videos, in which two damaged reef areas are shown, it is estimated that approximately about 90 and 60% of sponge individuals were broken down, respectively. Damaged areas showed an evident loss of tridimensional structure (Fig 5F). As the living tissue represents a minimal portion of the sponge body and the external surfaces of broken-down sponges are heavily covered by sediment, it was difficult to quantify live vs. dead individuals in those damaged areas. By conducting sporadic video zooms during the ROV transects, we corroborated that nearly all individuals laying on the bottom in an unnatural (i.e., non-erect) position had signs of necrosis (Fig 5E). Therefore, it was assumed that broken-down sponges will necessarily die in the long run.

**Fig 7:**
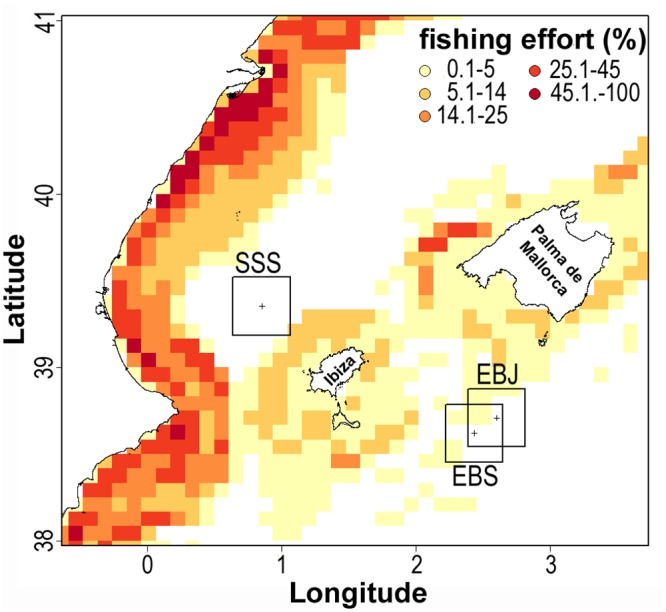
Spatial distribution of demersal fishing activities. Summary of a vessel monitoring system (VMS) analysis involving the fishing activity of 1045 vessels geared with otter trawls, set gillnets, set longlines, and/or traps during the 2007–2010 period. Results are expressed as relative (%) fishing effort (i.e., comparative numbers of fishing hours at a 5' x 5' grid cell). Crosses (+) refer to the location of the seamounts of interest (SSS, EBJ, EBS), each being surrounded by a 20 x 20 nautical mile quadrat.

The reasons for such a damage remain intriguing. We corroborated that fishing boats (> 15 m in length) geared with otter trawl, set gillnets, set longlines, and/or traps displayed intense activity along the continental margin of Spain and on the shelf and slope of the Balearic Islands during the 2007 to 2010 period (Fig 7). Nevertheless, no traceable fishing effort was recorded within a 20 nautical mile^-2^ area around the SSS site for the examined time period (Fig 7). It is likely that the fishing trip to this relatively offshore, small seamount was not economically profitable for either vessels based at the coast of Spain or for those at the Balearic Islands. Such an "in-between" geographical condition has probably favored the preservation of the lithistid formation so far. Yet, we detected a few fishing lines tangled around the sponges at SSS (S1 Fig), which could derive from fishing activities out of the 4-year time window investigated in this study or, more rarely, from fishing boats smaller than 15 m in length. In summary, it remains uncertain that recent fishing activities caused the documented damage at the SSS site. Some benthic fishing activity is detectable within the EBS-EBJ area. Yet it is moderate to low, representing fishing effort values lower than 5%.

A more serious threat is currently posed on the reef-like lithistid habitat by the plans of several international companies prospecting for gas and oil at extensive areas of the Balearic Sea, including the specific locations of SSS, EBS, and EBJ sites (S2–S3 Figs)

## Discussion

### Ecological significance

In the Mediterranean, lithistids can be said to be rare, with only 13 species (2.4%) out of the 533 living demosponges known to date in this sea. Such a well-known scarcity makes the occurrence of a Mediterranean deep aggregation exclusively built by the lithistid *L*. *pfeifferae* surprising. Even more surprising is that the species had never been described in the Mediterranean so far (see the section on "Taxonomic Remarks"), being only occasionally reported from bathyal depths at both sides of the Atlantic [31].

It remains unclear the particular conditions that have favored the impressive aggregation of *L*. *pfeifferae* at the SSS site and not at other seamounts of the Balearic Sea where the species has also been detected to occur (i.e., EBJ and EBS). As this sponge is characterized by a poorly developed organic body portion relative to a massive silica skeleton, it would be expected to require large amounts of dissolved silicon to growth and build its skeletal framework. However, average silicate concentrations at the -800 m isobath around the SSS area (about 8 μM over the year) were only 1 to 1.5 μM higher than values around the two others seamounts where the sponge occurs in far lower abundance. Concentrations at SSS are also consistently lower than those at the Atlantic side of the Gibraltar Strait, where the complex hydrodynamics makes silicate at the -800 m isobath to vary largely from around 10 to 17 μM over a year cycle, according to MEDAR data base. Concentrations at SSS are also far lower than average concentrations around the Pacific hexactinellid reefs at 200 m deep, typically being higher than 40 μM [17]. Altogether, these data suggest that: 1) dissolved silicon availability in the Balearic Sea may not be particularly responsible for the dense lithistid aggregation at SSS; 2) this lithistid sponge is able to build efficiently its massive silica skeleton in much lower silicate ambient concentrations than hexactinellid sponges, probably having a Si uptake system with efficient Si transport below the high silicate thresholds (100 to 200 μM) determined for other demosponges [6,41–42].

Regarding the others inorganic dissolved nutrients (ammonium, nitrate, nitrite and phosphate), temperature, and salinity, marked between-seamount differences did not occur. Food availability (i.e., bacterioplankton) is another major factor that could favor the sponge aggregation. Unfortunately, there are not detailed data around the SSS area relative to bacterial concentrations. It has been demonstrated that the concentration of the bathypelagic bacterioplankton has an enormous spatial variability that is coupled to complex local patterns of particulate organic carbon delivery from the euphotic zone [43]. The average concentration of Chl *a* at the -800 isobath around SSS site is 0.01 mg m^-3^ lower on average than concentrations at EBJ and EBS sites. Therefore, differences in concentration of sinking phytoplankton cannot account for the denser SSS aggregation. We have double-checked on this "food availability" hypothesis by examining dissolved oxygen values. The prokaryotic production and enzymatic activities in Mediterranean deep waters are among the highest reported worldwide at similar depths, indicating that the peculiar physical-chemical characteristics of the Mediterranean Sea, characterized by warm temperatures (typically 13°C around the studied seamount depths), would support high rates of organic carbon degradation and incorporation by prokaryotic assemblages [44]. Since prokaryotic production and enzymatic activities in deep water masses are known to be inversely related with oxygen concentration, we examined the -800 m isobath for drop anomalies in oxygen concentration, but, again, it varied minimally (0.12 ml/l) among the 3 seamounts over the year cycle (Fig 6C). In summary, from the available oceanographic data, we have detected only very minor between seamount differences, the magnitudes of which are too small to explain the astonishing dense lithistid aggregation at only SSS site but not at the two other seamounts where the species also occurs.

Interestingly, *L*. *pfeifferae* seems to be able to cope with heavy sediment deposition. In the particular zones where *L*. *pfeifferae* abounded at both SSS, EBJ, and EBS, the substratum was systematically covered by a thick sediment layer, suggesting that this sponge benefits somehow from regular and intense sediment deposition (S1–S3, S7 and S8 Videos). The location of the ostia on the silt-exposed side of the body, along with these ostia having a poral membrane (Fig 2H) that allows them to close, are two traits suggesting an adaption to cope with siltation and prevailing horizontal currents, and it may have favored the dominance of this particular sponge species at deep-water sites characterized by heavy sediment rain. While most other sponges minimize exposure to sediment [e.g., 45,46–47], the abundant needle-like spicules protruding at the inhalant side of the body and at the plate margin of *L*. *pfeifferae* (Fig 2G and 2H) appear to facilitate the retention and accumulation of sediment on the sponges (Fig 4C). These deposits of decaying silt may act as microorganism culturing medium, facilitating the proliferation of a self-regenerating bacterial community right on the feeding side of the sponge body, and it may represent an important food source to the sponges. To grow, these sponges may rely much on inputs of organic matter and dissolved silicon, the origin of which remains uncertain from the available oceanographic data. The interaction of seawater currents with the complex topography of seamounts usually creates intricate patterns of local circulation that may include both stationary ascending eddies (Taylor columns) and downstream eddies, along with trapped waves and internal wave reflection. Whatever the mechanism of food and silicate delivery to these lithistid populations is, it seems to happens in episodic pulses. This is deduced from the growth marks evident on the sponge bodies (Fig 5E), where the position of the free margin of the laminar body during successive growth stages can be easily retraced. Whether growth marks account for seasonal, annual, or other periodicity remains to be elucidated.

It cannot be ruled out that at least part of the development and/or maintenance of this sponge aggregation could result from a local hydrodynamic pattern around the SSS site avoiding gametes, embryos and/or larvae escaping from the parental habitat, and increasing local recruitment. Additionally, the SSS could function as concentration site for reproductive propagules emitted from other nearby sponge populations of the Balearic Sea. However, little more than mere speculation can be offered at the current stage of knowledge, because virtually nothing is known both about the local circulation around the SSS site and about the reproductive biology and dispersing ability of this group of lithistid sponges [48–49].

From our current, non-coring approach, it also remains unclear if the accumulation of buried skeletal silica frameworks makes a thick, true "bioherm", that is, with fossilized or sub-fossilized skeletal remains of dead individuals progressively accumulating while being buried by the sediment.

### Paleontological connections

Unequivocal fossil remains indicate that early representatives of hexactinellid and lithistid sponges occurred in the Cambrian (>540 mya). Soon lithistids became major reef framework builders (Early Ordovician; 485 mya) that irregularly expanded through the Paleozoic, while dictyonine hexactinellids did not build reefs until about 240 million years later, in the Mesozoic [18,50–55]. Although many of the Paleozoic lithistid and hexactinellid lineages did not make it through the Permian (252 mya) massive extinction [21,50–51], those few reaching the Mesozoic experienced a second major radiation and diversification through the Middle Jurassic. As a result, by the Late Jurassic (163–145 mya), sponge-dominated reefs thrived on continental shelves at varying depth within the photic layer, typically with the collaboration of cyanobacteria that produced a calcareous crust that further cemented the massive sponge silica skeletons [20,51,53,56–57]. On Late Jurassic and Early Cretaceous shelves (particularly at the "European" coasts of the Tethys Sea), hexactinellids and lithistids commonly co-occurred, often the former ones dominating in the deepest zones and the lithistids at the shallowest ones [20–21]. These sponge-dominated, silica reefs started declining in the Late Cretaceous (93 to 65 mya) and they progressively disappeared through the lower Tertiary (i.e., Paleogene: 65 to 23 mya) [20–21,53]. The most likely reason is that the evolutionary radiation of diatoms, coupled to a phenomenal ecological expansion and proliferation of this new group of silicon consumers, drastically decreased the availability of dissolved silicon in shallow water of the global ocean [58–59]. As a consequence, silicon became limiting to the sponges [60], with many reef building species becoming extinct and others migrating from the shelf to the aphotic slope, caves and other dark environments where diatoms could not exhaust dissolved silicon. Both the demise of sponge reefs and the bathymetrical migration of the hexactinellids and lithistids over the lower Tertiary are reflected in the fossil record [21,61–63]. The diatom-triggered silicate crisis also impacted the diversity and abundance of the siliceous lineages of radiolarians [64–65]. The fierce competition for dissolved silicon initiated around the Mesozoic-Tertiary boundary has reached Recent time and it is still affecting negatively the skeletal development of many modern shallow-water sponges, which outcompeted by diatoms, suffer a severe chronic silicon limitation [41–42,60]. Such a persisting silicon limitation would probably prevent sponge silica reefs and other silica-rich sponge aggregations to re-conquer the photic zone, even if increasing ocean acidification will negatively affect the viability of coral reefs in future oceans.

It is worth noting that the general lithistid lineage to which *Leiodermatium* belongs (palaeontologically referred to as "Rhizomorina" and characterized by rhizoclone desma) has survived the amazing journey from the Cambrian to Recent time. Remains of the genus *Leiodermatium* can be traced back with reliability to the middle Jurassic, as proved by the occurrence of the bodily preserved *Leiodermatium* (originally described as *Azorica*) *calloviensis* [21,66–67]. Therefore, because members of the genus *Leiodermatium* were already part of the Mesozoic sponge-dominated reefs, the uncovered *Leiodermatium* reef-like formation at SSS is foreseen as a unique living laboratory. The identification of the geomorphological, oceanographic and biological circumstances that favored and maintain such a modern lithistid aggregation may teach us about the contribution of lithistid sponges to the development and functioning of the Mesozoic reefs.

### Taxonomic remarks

With relatively few morphological and skeletal characters available in the genus *Leiodermatium* to discriminate among species, *Leiodermatium pfeifferae* (Carter, 1873) [68–69] and *Leiodermatium lynceous* Schmidt, 1870 [70] are currently regarded as distinguishable, but very similar species. They also have largely overlapping bathymetrical and distribution ranges. *Leiodermatium pfeifferae* has been reported from both sides of the Atlantic (Brazil, Portugal, Açores, Madeira) and in the Western Mediterranean (this work). Likewise, *L*. *lynceous* has been reported from both sides of the Atlantic (Brazil, Caribbean, England coasts, Portugal, Strait of Gibraltar, Canary Islands) and in the Mediterranean (Tyrrhenian [71], the Ionian [72], and the Aegean [73] Sea).

Whether these two species are indeed valid and distinguishable from each other, it has been questioned from the very first description of *L*. *pfeifferae* by Carter in 1873 [68–69] until the last review of the genus by Kelly in 1997 [31]; some authors have also regarded them as synonyms [71]. At present, the species discrimination could be said to rely on several characters of arguable phylogenetic value. Unlike *L*. *lynceus*, *L*. *pfeifferae* bears the oscula on the concave side of the plates, makes thicker (>4 mm) and larger plates, and has more abundant needle-like spicules (oxeas), making a microscopically hispid fringe at the margin of the plates. In this regard, our collected individuals, having the oscula on the concave side (Fig 2C), forming thick plates (4 to 9 mm), and with abundant oxeas (Fig 3A and 3B) at the inhalant side and the marginal fringe (Fig 2G and 2H), match completely the description of the holotype of *L*. *pfeifferae* by Carter [68–69]. Additionally, Carter [69] described the "oxeas" of *L*. *pfeifferae* being up to 1,800 μm long and x 8.5 μm thick, noting that the largest ones, typically at the marginal fringe, were not oxeas, but strongyles or strongyloxeas, because the acerate points became round, slightly inflated ends that could even bear tiny spines. Those details, which have not been reported from specimens of *L*. *lynceus* so far, do also occur in the material that we collected from SSS site (Fig 3C and 3D). We have found that the oxeas hispidating the inhalant side of the sponges are very thin (1–2 μm) and flexuous (Figs 2G, 3A, 3B, 3E, and 3G), while those making the marginal fringe of the plate are thicker (up to 8 μm), less flexuous, and with slightly spiny round ends, that is, becoming strongyloxeas or strongyles (Fig 3C and 3D). We also noticed that these oxeas and strongyloxeas have a triangular axial canal at their core (Fig 3F), while no axial canal was ever found within the arms of broken rhizoclone desmata (Fig 3G and 3H).

### Conservation concerns

The large, erect, plate-like bodies of *L*. *pfeifferae* are seen as particularly susceptible to be broken by tangles with fishing gear and telecom cables, but also by landing incorrectly ROVs and deploying benthic scientific or mining equipment. Neither can aggressive seismic exploration be ruled out as a putative damaging factor. Because deep-sea erect, plate-like sponges are often suited to exploit horizontal prevailing currents, the individuals that are broken and fall to the bottom in an unnatural position are thought to have minimum chances of surviving. Therefore, the architectural organization of this sponge aggregation makes it particularly vulnerable to physical damage. It is worth noting that, according to the VMS data, the area where the densest lithistid aggregation is known (SSS) does not appear to be a significant target of the demersal fishing fleet. Nevertheless, a few fishing lines were found tangled around the sponges (S1 Fig) and ample areas with detached and broken sponges were noticed as well (S7 and S8 Videos). The latter finding indicates that some human or natural agent or process, unidentified from the current approach, is already damaging this singular lithistid formation.

The glass sponge reefs in the Canadian Pacific and, in general, sponge aggregations on deep shelves and slopes are currently recognized as essential marine habitats, with growing concerns over the need for effective and urgent preservation [5,11,14,28,74–77]. Currently, when seamounts are becoming more attractive and accessible to benthic fishing [78–79], and when the Mediterranean continental margin of Spain is being targeted for extensive drilling, prospection aimed at commercial gas hydrate extraction, fracking of natural gas and oil, and mineral mining [80–81], urgent conservation measures are required to prevent further damage of the newly discovered lithistid aggregation. More specifically, the imminent plans for extraction of gas and oil in extensive areas of the Balearic Sea, including the three studied seamounts pose a serious, major threat on the discovered lithistid aggregations (S2–S3 Figs). It would be regrettable to have the singular habitat at SSS altered or destroyed even before it can be studied in detail and its lessons to the past extracted.

## Supporting Information

S1 Fig Examples of fishing lines tangled on the sponges. (A-B) Fishing lines tangled around some of the sponges were documented. Fishing lines were often buried in the sediment, and mostly noticed by the lineal marks (arrows) they left on the bottom around the sponges.
(TIF)

S2 Fig Map summarizing current projects for prospection and exploitation of bathyal bottoms at the Balearic Sea. The map has been elaborated using public information available from the Spanish Ministry of Industry, Energy and Tourism, published in the Spanish Boletín Oficial del Estado (BOE) and at the Ministry webpage: http://www6.mityc.es/aplicaciones/energia/hidrocarburos/petroleo/exploracion2014/mapas/inicio.html. The company Spectrum Geo Limited has requested permission (i.e., Spectrum project) to the Spanish Government to conduct seismic survey of extensive bathyal bottoms aimed to a subsequent exploitation of hydrocarbon deposits. The activity will be conducted in two phases, with stage 2 (green grid) involving the areas where EBJ and EBS seamounts are located. The company Cairn Energy has requested permission (i.e., Cairn project) to seismic prospecting, to research, and to extract hydrocarbons at an area of the Balearic Sea that includes the SSS location, that is, the seamount where the unique lithistid reef-like aggregation occurs (see a detail of the Cairn project in S3 Fig). The global impact area is estimated as an outer, 30 km-wide belt around the zone of activity.
(TIF)

S3 Fig Summary of permits requested to conduct deep-sea activities in the vicinity of the SSS habitat. The map shows the area requested for a variety of planned activities by the enterprise Cairn Energy. It also shows the location of relevant topography features and benthic communities, including the singular lithistid reef at SSS. The global impact area is estimated as an outer, 30 km-wide belt around the zone of activity. The map has been elaborated using public information available from the Spanish Ministry of Industry, Energy and Tourism, published in the Boletin Oficial del Estado (BOE) and at Ministry the web page: http://www6.mityc.es/aplicaciones/energia/hidrocarburos/petroleo/exploracion2014/mapas/inicio.html
(TIF)

S1 Video Views of the lithistid reef at SSS. (AVI)

S2 Video Views of the lithistid reef at SSS. (AVI)

S3 Video Views of the lithistid reef at SSS. (AVI)

S4 Video Views of epibiont fauna on a lithistid sponge. (AVI)

S5 Video Views of benthic macrofauna associated to the reef. (AVI)

S6 Video Views of benthic macrofauna associated to the reef. (AVI)

S7 Video Views of a damaged reef area 1 at SSS. (AVI)

S8 Video Views of a damaged reef area 2 at SSS. (AVI)

## References

[1] Reiswig HM. Water transport, respiration and energetics of three tropical marine sponges. J Exp Mar Biol Ecol. 1974;14: 231–249.

[2] Pile AJ, Young CM. The natural diet of a hexactinellid sponge: Benthic-pelagic coupling in a deep-sea microbial food web. Deep-Sea Res. 2006;I 53: 1148–1156.

[3] Bell JJ. The functional roles of marine sponges. Estuar Coast Shelf Sci. 2008;79: 341–353.

[4] Buhl-Mortensen L, Vanreusel A, Gooday AJ, Levin LA, Priede IG, Buhl-Mortensen P, et al Biological structures as a source of habitat heterogeneity and biodiversity on the deep ocean margins. Mar Ecol. 2010;31: 21–50.

[5] Hogg MM, Tendal OS, Conway KW, Pomponi SA, van Soest RWM, Gutt J, et al Deep-Sea sponge grounds: Reservoirs of biodiversity. Cambridge: UNEP-WMCM; 2010.

[6] Maldonado M, Ribes M, Van Duyl FC. Nutrient fluxes through sponges: Biology, budgets, and ecological implications. Adv Mar Biol, 2012;62: 114–182. 10.1016/B978-0-12-394283-8.00003-5 22664122

[7] Rützler K. The role of sponges in the Mesoamerican barrier-Reef ecosystem, Belize. Adv Mar Biol. 2012;61: 211–271. 10.1016/B978-0-12-387787-1.00002-7 22560779

[8] de Goeij JM, van Oevelen D, Vermeij MJA, Osinga R, Middelburg JJ, de Goeij AFPM, et al Surviving in a marine desert: The sponge loop retains resources within coral reefs. Science. 2013;342: 108–110. 10.1126/science.1241981 24092742

[9] Rice AL, Thurston MH, New AL. Dense aggregations of a hexactinellid sponge, *Pheronema carpenteri*, in the Porcupine Seabight (northeast Atlantic Ocean), and possible causes. Prog Oceanogr. 1990;24: 179–196.

[10] Beaulieu SE. Life on glass houses: Sponge stalk communities in the deep sea. Mar Biol. 2001;138: 803–817.

[11] Klitgaard AB, Tendal OS. Distribution and species composition of mass occurrences of large-sized sponges in the northeast Atlantic. Prog Oceanogr. 2004;61: 57–98.

[12] Maldonado M, Carmona MC, Velásquez Z, Puig A, Cruzado A, López A, et al Siliceous sponges as a silicon sink: An overlooked aspect of the benthopelagic coupling in the marine silicon cycle. Limnol Oceanogr. 2005;50: 799–809.

[13] Bo M, Bertolino M, Bavestrello G, Canese S, Giusti M, Angiolillo M, et al Role of deep sponge grounds in the Mediterranean Sea: A case study in southern Italy. Hydrobiol. 2012; 687: 163–177.

[14] Levin LA, Sibuet M. Understanding continental margin biodiversity: A new Imperative. Ann Rev Mar Sci. 2012;4: 79–112. 22457970 10.1146/annurev-marine-120709-142714

[15] Knudby A, Kenchington E, Murillo FJ. Modeling the distribution of *Geodia* sponges and sponge grounds in the Northwest Atlantic. PLoS One. 2013;8: e82306 10.1371/journal.pone.0082306 24324768 PMC3852940

[16] Conway KW, Barrie JV, Austin WC, Luternauer JL. Holocene sponge bioherms on the western Canadian continental shelf. Cont Shelf Res. 1991;11: 771–790.

[17] Chu JWF, Maldonado M, Yahel G, Leys SP. Glass sponge reefs as a silicon sink. Mar Ecol Prog Ser. 2011;441: 1–14.

[18] Krautter M, Conway KW, Barrie JV, Neuweiler M. Discovery of a "living dinosaur": Globally unique modern hexactinellid sponge reefs off British Columbia, Canada. Facies. 2001;44: 265–282.

[19] Conway KW, Barrie JV, Krautter M. Geomorphology of unique reefs on the western Canadian shelf: Sponge reefs mapped by multibeam bathymetry. Geo-Mar Lett. 2005;25: 205–213.

[20] Ghiold J. The sponges that spanned Europe. New Sci. 1991;129: 58–62.

[21] Wiedenmayer F. Contributions to the knowledge of post-Paleozoic neritic and archibental sponges (Porifera). Schweiz Paläontol Abhand. 1994;116: 1–147. 11563352

[22] Lévi C. Lithistid Sponges from the Norfolk Rise. Recent and Mesozoic Genera In: Reitner J, Keupp H, editors. Fossil and Recent Sponges. New York: Springer Verlag;1991 pp. 72–82.

[23] Reid RE. Tethys and the zoogeography of some modern and Mesozoic Porifera. Publ Syst Assoc. 1967;7: 171–181.

[24] Lévi C, Lévi P. Eponges Tétractinellides et Lithistides bathyales de Nouvelle-Calédonie. Bull Mus Natl Hist Nat Paris. 1983; 4eme série, 5: 101–168.

[25] Whitney F, Conway K, Thomson R, Barrie V, Krautter M, Mungov G. Oceanographic habitat of sponge reefs on the Western Canadian Continental Shelf. Cont Shelf Res. 2005;25(2): 211–226.

[26] Conway KW, Barrie JV, Hill PR, Austin WC, Pickard K. Mapping sensitive benthic habitats in the Strait of Georgia, coastal British Columbia: Deep-water sponge and coral reefs. Geol Surv Can. 2007;A2: 1–6.

[27] Stone RP, Conway KW, Csepp DJ, Barrie JV. The boundary reefs: Glass sponge (Porifera: Hexactinellidae) reefs on the international border between Canada and the United States NOAA Technical Memorandum NMFS-AFSC-264; 2014.

[28] Cook SE, Conway KW, Burd B. Status of the glass sponge reefs in the Georgia Basin. Mar Environ Res. 2008;66, Supplement: S80–S86. 10.1016/j.marenvres.2008.09.002 18954900

[29] Kahn AS, Yahel G, Chu JWF, Tunnicliffe V, Leys SP. Benthic grazing and carbon sequestration by deep-water glass sponge reefs. Limnol Oceanogr. 2015;60,1: 78–88.

[30] Kelly M. Description of a new lithistid sponge from northeastern New Zealand, and consideration of the phylogenetic affinities of families Corallistidae and Neopeltidae. Zoosystema. 2000;22: 265–283.

[31] Kelly M. The Marine Fauna of New Zealand. Porifera: Lithistid Demospongiae (Rock Sponges) Wellington: National Institute of Water and Atmospheric Research (NIWA); 2007.

[32] Pomponi SA, Kelly M, Reed J, Wright AD. Diversity and bathymetric distribution of lithistid sponges in the tropical western Atlantic region. Bull Biol Soc Wash. 2001;10: 344–353.

[33] Manconi R, Serusi A. Rare sponges from marine caves: Discovery of *Neophrissospongia nana* nov. sp. (Demospongiae, Corallistidae) from Sardinia with an annotated checklist of Mediterranean lithistids. ZooKeys. 2008;4: 71–87.

[34] Pisera A, Vacelet J. Lithistid sponges from submarine caves in the Mediterranean: Taxonomy and affinities. Sci Mar. 2011;75: 17–40.

[35] Lévi C, Lévi P. Spongiaires (MUSORSTOM 1 & 2)—Résultats des Campagnes MUSORSTOM. Mém Mus Natl Hist Nat. 1989;(A, Zoologie), 143: 25–103.

[36] Lévi C. Porifera Demospongiae: Spongiaires bathyaux de Nouvelle-Calédonie, récoltés par le ‘Jean Charcot’. Campagne BIOCAL,1985. Mém Mus Natl Hist Nat. 1993;(A, Zoologie), 158: 1–426.

[37] Schlacher-Hoenlinger MA, Pisera A, Hooper JNA. Deep-sea "lithistid" assemblages from Norfolk Ridge (New Caledonia), with description of seven new species and a new genus (Porifera, Demospongiae). Zoosystema. 2005;27: 649–698.

[38] Lee J, South AB, Jennings S. Developing reliable, repeatable, and accessible methods to provide high-resolution estimates of fishing-effort distributions from vessel monitoring system (VMS) data. ICES J Mar Sci. 2010;67: 1260–1271.

[39] Hintzen NT, Bastardie F, Beare D, Piet GJ, Ulrich C, Deporte N, et al VMStools: Open-source software for the processing, analysis and visualisation of fisheries logbook and VMS data. Fish Res. 2012;115–116: 31–43.

[40] Acosta J, Ancochea E, Canals M, Huertas MJ, Uchupi E. Early Pleistocene volcanism in the Emile Baudot Seamount, Balearic Promontory (western Mediterranean Sea). Mar Geol. 2004;2007: 247–257.

[41] Frøhlich H, Barthel D. Silica uptake on the marine sponge *Halichondria panicea* in Kiel Bight. Mar Biol. 1997;128: 115–125.

[42] Maldonado M, Navarro L, Grasa A, González A, Vaquerizo I. Silicon uptake by sponges: A twist to understanding nutrient cycling on continental margins. Sci Rep. 2011;1: 1–8. 10.1038/srep00001 22355549 PMC3216517

[43] Hansell DA, Ducklow HW. Bacterioplankton distribution and production in the bathypelagic ocean: Directly coupled to particulate organic carbon export? Deep-Sea Res I. 2003;48: 150–156.

[44] Luna GM, Bianchelli S, Decembrini F, De Domenico E, Danovaro R, Dell'Anno A. The dark portion of the Mediterranean Sea is a bioreactor of organic matter cycling. Global Biogeochem Cycles. 2012;26: GB2017.

[45] Maldonado M, Young CM. Bathymetric patterns of sponge distribution on the Bahamian slope. Deep-Sea Res I. 1996;43: 897–915.

[46] Maldonado M, Uriz MJ. An experimental approach to the ecological significance of microhabitat-scale movement in an encrusting sponge. Mar Ecol Prog Ser. 1999;185: 239–255.

[47] Elliott GRD, Leys SP. Coordinated contractions effectively expel water from the aquiferous system of a fresh water sponge. J Exp Biol. 2007;2010: 3736–3748. 10.1242/jeb.003392 17951414

[48] Maldonado M. The ecology of the sponge larva. Can J Zool. 2006;84: 175–194.

[49] Maldonado M, Riesgo A. Reproduction in the phylum Porifera: A synoptic overview. Treb Soc Cat Biol. 2008;59: 29–49.

[50] Finks RM. Late Paleozoic sponge faunas of the Texas region: The siliceous sponges. Bull Am Mus Nat Hist N Y. 1960;120: 1–160.

[51] Wiedenmayer F. Siliceous sponges In: Hartman WD, Wendt JW, Wiedenmayer F, editors. Living and fossil sponges Notes for a short course. Miami: University of Miami; 1980 pp. 55–85.

[52] Johns RA. Ordovician lithistid sponges of the Great Basin Austin: Nevada Bureau of Mines and Geology; 1994.

[53] Kauffman EG, Herm D, Johnson CC, Harries P, éFling RH. The ecology of Cenomanian lithistid sponge frameworks, Regensburg area, Germany. Lethaia. 2000; 33: 214–235.

[54] Adachi N, Ezaki Y, Liu J. Early Ordovician shift in reef construction from microbial to metazoan reefs. Palaios. 2011;26: 106–114.

[55] Antcliffe JB, Callow RHT, Brasier MD. Giving the early fossil record of sponges a squeeze. Biol Rev. 2014;89: 972–1004. 10.1111/brv.12090 24779547

[56] Gygi R. Eustatic sea level changes of the Oxfordian (Late Jurassic) and their effect documented in sediments and fossil assemblages of an epicontinental sea. Eclogae Geol Helv. 1986; 79: 455–491.

[57] Werner W, Leinfelder RR, Fürsich FT, Krautter M. Comparative palaeoecology of marly coralline sponge-bearing reefal associations from the Kimmeridgian (Upper Jurassic) of Portugal and Southwestern Germany. Cour Forschungsinst Senckenb. 1994;172: 381–397.

[58] Maliva RG, Knoll AH, Siever R. Secular change in chert distribution: A reflection of evolving biological participation in the silica cycle. Palaios. 1989;4: 519–532. 11539810

[59] Siever R. Silica in the oceans: Biological-geochemical interplay In: Scheneider SH, Boston PJ, editors. Scientists on Gaia. Cambridge, MA: MIT Press; 1991 pp. 287–295.

[60] Maldonado M, Carmona MC, Uriz MJ, Cruzado A. Decline in Mesozoic reef-building sponges explained by silicon limitation. Nature. 1999;401: 785–788.

[61] Moret L.Contribution à l'étude des spongiaires siliceux du Miocene de l'Algerie. Mém Soc Geol Fr. 1924;1: 1–27.

[62] Moret L. Contribution à l'étude des Spongiaires siliceus du Crétacé supérieur français. Mém Soc Geol Fr. 1925;5: 1–303.

[63] Moret L. Contribution à l’étude des spongiaires siliceux du Crétacé supérieur français. Mém Soc Geol Fr. 1926;5: 1–327.

[64] Lowenstam HA, Weiner S. On biomineralization New York: Oxford University Press; 1989.

[65] Lazarus DB, Kotrc B, Wulf G, Schmidt DN. Radiolarians decreased silicification as an evolutionary response to reduced Cenozoic ocean silica availability. Proc Natl Acad Sci USA. 2009; 106: 9333–9338. 10.1073/pnas.0812979106 19458255 PMC2695065

[66] Moret L. Les Spongiaires siliceux du Callovien de la Voultesur-Rhône (Archède). Trav Lab Géol Fac Sci Lyon. 1928;13: 123–140. 3770985

[67] Charbonnier S, Vannier J, Gaillard C, Bourseau J-P, Hantzpergue P. The La Voulte Lagerstätte (Callovian): Evidence for a deep water setting from sponge and crinoid communities. Palaeogeogr, Palaeoclimatol, Palaeoecol. 2007;250: 216–236.

[68] Carter HJ. On the Hexactinellidae and Lithistidae generally, and particularly on the Aphrocallistidae, Aulodictyon, and Farreae, together with facts elicited from their feciduous structures, and descriptions repectively of three new species. Ann Mag Nat Hist. 1873;4th series, 12: 349–472.

[69] Carter HJ. Descriptions and figures of deep-sea sponges and their spicules, from the Atlantic Ocean, dredged up on board H.M.S. "Porcupine", chiefly in 1869 (concluded). Ann Mag Nat Hist. 1876;4th series, 18: 225–479.

[70] Schmidt O. Grundzüge einer Spongien-Fauna des Atlantischen Gebietes. Leipzig: Engelmann; 1870.

[71] Magnino G, Gravina MF, Righini P, Serena F, Pansini M. Due demosponge Lithistidi nuove per i mari italiani. Biol Mar Mediterr. 1999;6: 391–393.

[72] Longo C, Mastrototaro F, Corriero G. Sponge fauna associated with a Mediterranean deep-sea coral bank. J Mar Biol Assoc UK. 2005;85: 1341–1352.

[73] Vamvakas CN. Contribution in the study of soft substrata benthic communities in the Hellenic Seas, West Saronikos Gulf area. Hellenic Ocean and Limnol. 1971;20: 129–272.

[74] UNEP. Ecosystems and Biodiversity in Deep Waters and High Seas. Switzerland: United Nations Environment Programme / World Conservation Union; 2006.

[75] Samadi S, Schlacher TA, Richer de Forges B. Seamount benthos In: Pitcher TJ, Morato T, Hart PJB, Clark MR, Haggan N, Santos RS, editors. Seamounts: Ecology, Fisheries & Consevation. Oxford: Blackwell Publishing; 2007 pp. 119–140. 10.1371/journal.pone.0095839

[76] FAO. Workshop for the Development of a Global Database for Vulnerable Marine Ecosystems (VMEs). Rome: FAO 1018; 2013.

[77] Kenchington E, Murillo FJ, Lirette C, Sacau M, Koen-Alonso M, Kenny A, et al Kernel density surface modelling as a means to identify significant concentrations of vulnerable marine ecosystem indicators. PLoS One. 2014;9: 1–14. 10.1371/journal.pone.0109365 PMC4188592 25289667

[78] Watson R, Kitchingman A, Cheung WW. Catches from the world seamount fisheries In: Pitcher TJ, Morato T, Hart PJB, MR, Haggan N et al, editors. Seamounts: Ecology, fisheries & conservation. Oxford: Blackwell Publishing; 2007 pp. 400–412. 10.1371/journal.pone.0095839

[79] Clark MR, Koslow JA. Impacts of fisheries on seamounts In: Pitcher TJ, Morato T, Hart PJB, Clark MR, Haggan N, Santos RS, editors. Seamounts: Ecology, fisheries & conservation. Oxford: Blackwell Publishing;2007 pp. 413–441. 10.1371/journal.pone.0095839

[80] Glasby GP. Potential impact on climate of the exploitation of methane hydrate deposits offshore. Mar Pet Geol. 2003; 20: 163–175.

[81] Milkov AV. Global estimates of hydrate-bound gas in marine sediments: how much is really out there? Earth-Sci Rev. 2004;66: 183–197.

