# Supplementary figures and images for: Aggregated Clumps of Lithistid Sponges: A Singular, Reef-Like Bathyal Habitat with Relevant Paleontological Connections
Source: PLoS One. 2015 May 27;10(5):e0125378. doi: 10.1371/journal.pone.0125378 (PMC4446211; doi:10.1371/journal.pone.0125378)

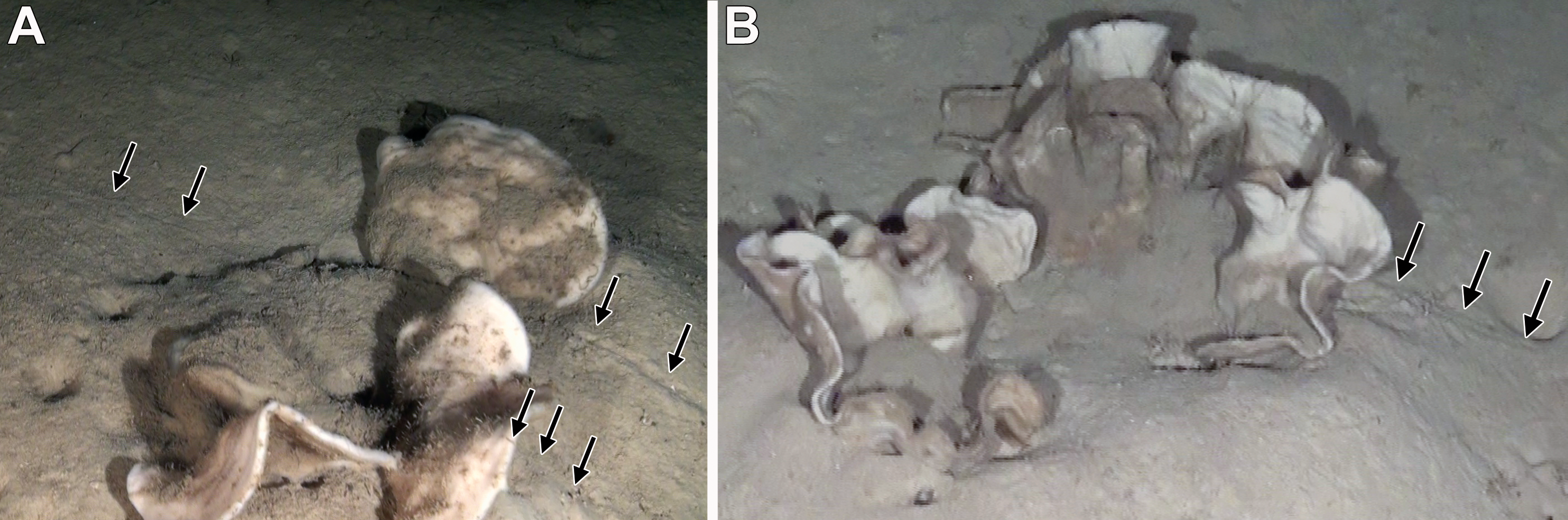

Supplement: S1 Fig — (A-B) Fishing lines tangled around some of the sponges were documented. Fishing lines were often buried in the sediment, and mostly noticed by the lineal marks (arrows) they left on the bottom around the sponges. (TIF) [file pone.0125378.s001.tif]

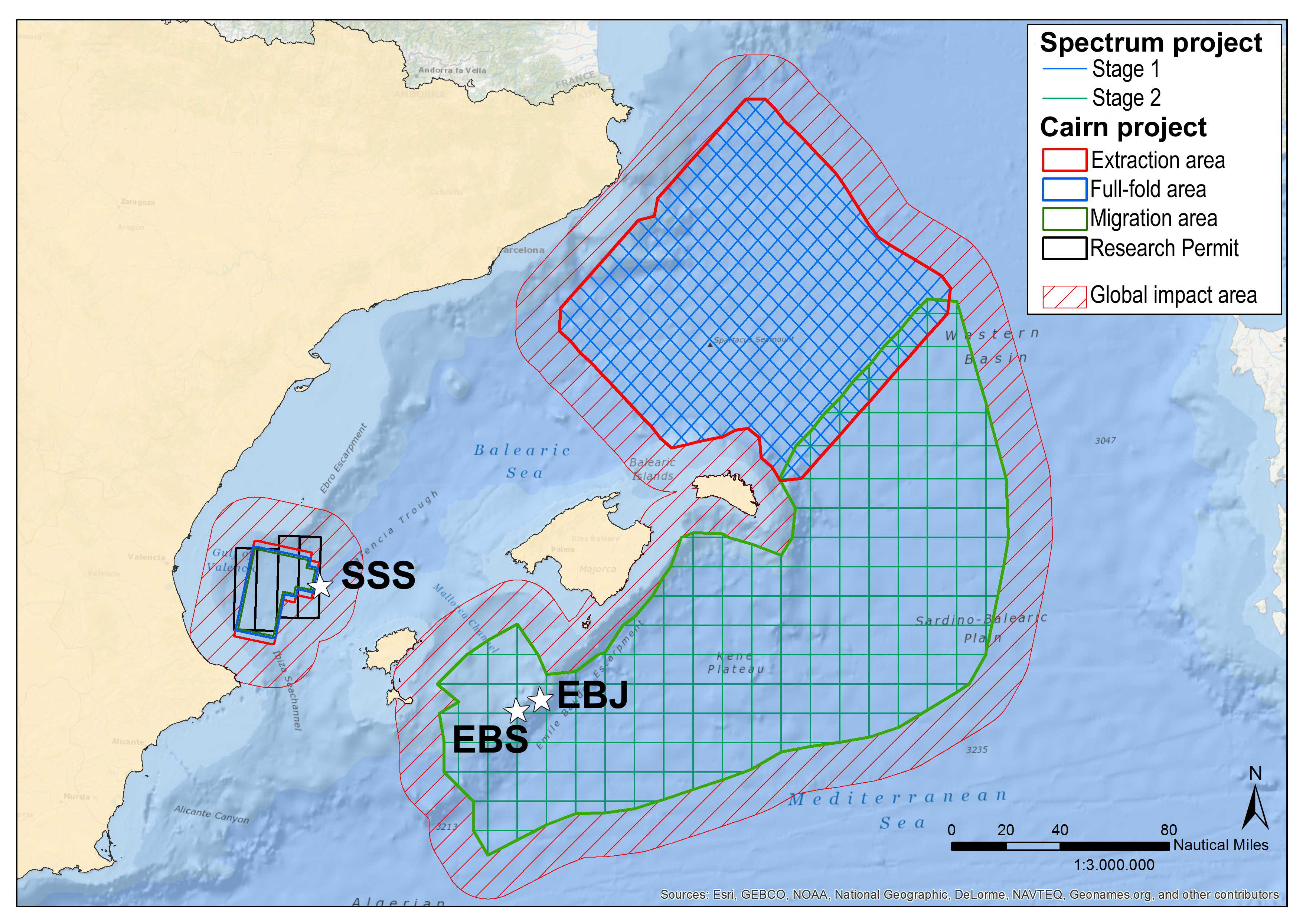

Supplement: S2 Fig — The map has been elaborated using public information available from the Spanish Ministry of Industry, Energy and Tourism, published in the Spanish Boletín Oficial del Estado (BOE) and at the Ministry webpage: http://www6.mityc.es/aplicaciones/energia/hidrocarburos/petroleo/exploracion2014/mapas/inicio.html. The company Spectrum Geo Limited has requested permission (i.e., Spectrum project) to the Spanish Government to conduct seismic survey of extensive bathyal bottoms aimed to a subsequent exploitation of hydrocarbon deposits. The activity will be conducted in two phases, with stage 2 (green grid) involving the areas where EBJ and EBS seamounts are located. The company Cairn Energy has requested permission (i.e., Cairn project) to seismic prospecting, to research, and to extract hydrocarbons at an area of the Balearic Sea that includes the SSS location, that is, the seamount where the unique lithistid reef-like aggregation occurs (see a detail of the Cairn project in S3 Fig). The global impact area is estimated as an outer, 30 km-wide belt around the zone of activity. (TIF) [file pone.0125378.s002.tif]

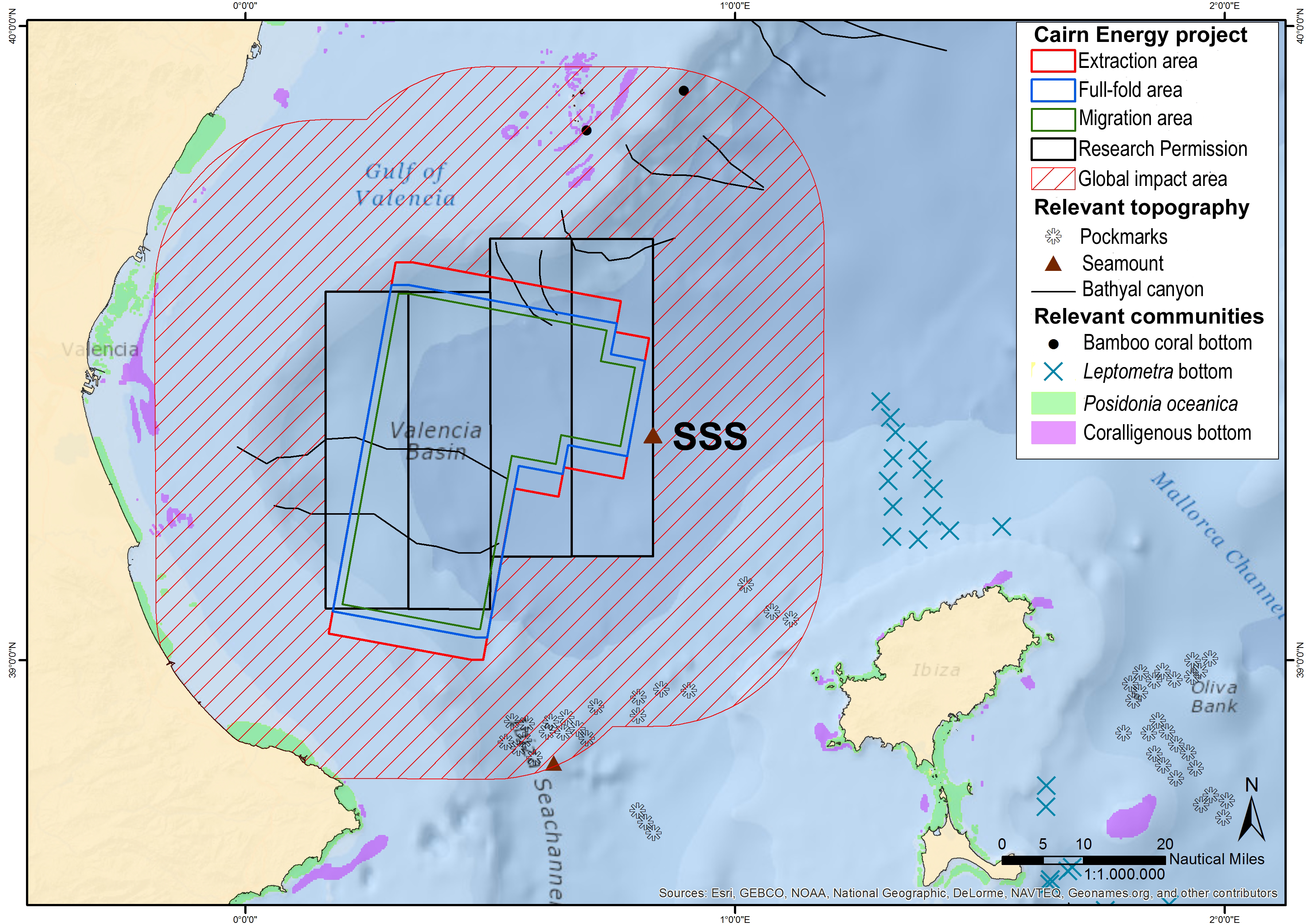

Supplement: S3 Fig — The map shows the area requested for a variety of planned activities by the enterprise Cairn Energy. It also shows the location of relevant topography features and benthic communities, including the singular lithistid reef at SSS. The global impact area is estimated as an outer, 30 km-wide belt around the zone of activity. The map has been elaborated using public information available from the Spanish Ministry of Industry, Energy and Tourism, published in the Boletin Oficial del Estado (BOE) and at Ministry the web page: http://www6.mityc.es/aplicaciones/energia/hidrocarburos/petroleo/exploracion2014/mapas/inicio.html (TIF) [file pone.0125378.s003.tif]
